# Positive DAT-SCAN in SPG7: a case report mimicking possible MSA-C

**DOI:** 10.1186/s12883-021-02345-y

**Published:** 2021-08-25

**Authors:** Gabriele Bellini, Eleonora Del Prete, Elisa Unti, Daniela Frosini, Gabriele Siciliano, Roberto Ceravolo

**Affiliations:** 1 Department of Clinical and Experimental Medicine, Unit of Neurology, University of Pisa, Pisa, Italy; 2 Department of Medical Specialties, Neurology Unit, AOUP, Pisa, Italy

**Keywords:** SPG7, DAT-SCAN imaging, Multiple system atrophy, Nigrostriatal denervation, Case report

## Abstract

**Background:**

Spastic Paraplegia type 7 (SPG7) is one of the most common autosomal recessive Hereditary Spastic Paraplegias (HSP); Spastic Paraplegias (SPGs) can present as hereditary ataxias. However, ataxia is frequently the symptom of presentation of many other hereditary/sporadic disorders, such as Multiple system atrophy type C (MSA-C), an α-synuclein sporadic neurodegenerative disorder, in which cerebellar ataxia is one of the main clinical features. Dopamine Transporter imaging (DAT-SCAN), associated with clinical features, can be a helpful tool in order to distinguish MSA-C from other causes of ataxia.

**Case-presentation:**

We present the case of a 70-year-old man with gait difficulties over a period of 3 years and frequent backward/lateral falls. He also reported urinary urge incontinence, but no symptoms that are compatible with orthostatic hypotension. On neurological examination he showed ataxic gait, spasticity in the left lower limb and trunk and limb ataxia, especially on the left side. Mild hypokinesia was found in all 4 limbs, especially in the left foot. MRI revealed atrophy of the cerebellar hemispheres and vermis. DAT-SCAN imaging revealed bilateral nigro-striatal degeneration, which was compatible with a diagnosis of possible MSA-C. Considering the atypical disease course (the patient walked without any support after 3 years), we carried out a genetic investigation for Ataxia, and a mutation in *SPG7* was found.

**Conclusions:**

DAT-SCAN imaging, evaluated together with the clinical findings, can be useful for differentiating MSA from other possible causes of adult-onset Ataxia. Indeed, patients with MSA-C generally show a decreased uptake of dopamine transporters in DAT-SCAN imaging. Ours is the first case reported in the literature of a patient with *SPG7* mutation with nigrostriatal degeneration and a clinical presentation of a possible MSA-C. Performing genetic investigations in patients with an atypical disease course is important to avoid MSA-mimicries.

Identifying the correct diagnosis is important not only for prognostic reasons, but also for possible future genetic therapies.

## Background

Adult-onset ataxias have been defined as ataxic syndromes which begin after the age of 40 [1]. In a practical diagnosis approach, adult-onset ataxias can be classified in three major groups: non-hereditary degenerative ataxias, acquired ataxias and hereditary ataxias [2].

Among genetic forms of ataxia we can distinguish autosomal dominant forms, spinocerebellar ataxia (SCA), which is associated with trinucleotide repeat expansions in most cases; autosomal recessive forms, such as Friedreich Ataxia caused by intronic GAA repeat expansions of the *FXN* gene; sensory ataxic neuropathy, dysarthria and ophthalmoparesis syndrome (SANDO) caused by *POLG* mutations, Ataxia telangiectasia caused by *ATM* mutations or the Niemann-Pick disease type C caused by *NPC1* or *NPC2* mutations; X linked Ataxia, such as the fragile X tremor-ataxia syndrome caused by CGG repeat expansions within the *FMR1* gene; Mitochondrial Ataxia, that includes Kearns-Sayre syndrome, myoclonic epilepsy with ragged red fibers (MERRF), mitochondrial encephalopathy, lactic acidosis, and stroke-like episodes (MELAS) [2].

Spastic Paraplegias (SPGs) can present as hereditary ataxias [3]. Hereditary Spastic Paraplegias (HSP) are characterized by progressive spasticity and weakness of the lower limbs, caused by a degenerative axonopathy of the corticospinal tract. Urinary urgency and a mild impairment of deep sensory modalities are sometimes reported [4].

Spastic paraplegia type 7 (SPG7) is one of the most common autosomal recessive HSPs, characterized by insidiously progressive bilateral lower-limb weakness and spasticity. Ataxia can be present in up to 57% of patients and cerebellar atrophy can be detected on MRI in one-third of cases [5]. Other common symptoms may be spastic dysarthria, dysphagia, ophthalmic findings (nystagmus, strabismus, ptosis, pale optic disks) and urinary sphincter disturbances [5].

One of the most common causes of non-hereditary degenerative ataxia is MSA-C, but it is important to consider that sporadic cases of hereditary ataxia are not infrequent [6].

Multiple system atrophy (MSA) is an α-synuclein sporadic neurodegenerative disorder with a mean age at onset of 54 years [7]. The main clinical features of MSA are autonomic failure, parkinsonism, cerebellar ataxia and pyramidal tract signs in various combinations [7]. On the basis of the predominant symptomatology, a parkinsonian (MSA-P) or cerebellar (MSA-C) subtype has been described [8, 9].

Recently, Cortese and colleagues identified a recessive biallelic repeat expansion, (AAGGG) exp., in the *RFC1* gene that may be responsible for 90% of cases with full-blown cerebellar ataxia, neuropathy and vestibular areflexia syndrome (CANVAS) [10]. Moreover, Wan and colleagues identified biallelic (AAGGG) exp. in three MSA patients, raising the possibility that MSA might share the same genetic background as CANVAS [11].

In the literature, cases of misdiagnosis between SPG7 and MSA-C have been reported, probably because of the presence of some common features, such as ataxia or autonomic disfunction [12].

We describe the case of a patient with a clinical diagnosis of a possible MSA-C with nigrostriatal degeneration on Dopamine Transporter imaging (DAT-SCAN), and in whom *SPG7* mutations were detected in the genetic analysis. The patient’s informed written consent was obtained for the publication of his clinical data.

## Case presentation

A 70-year-old man was referred to the Movement Disorder Unit of the University of Pisa for gait difficulties, described as the tendency towards a left lateropulsion while walking followed by frequent backward/lateral falls and difficulty in standing up from a sitting position. This had lasted for 3 years, and in the same period he also referred difficulty in controlling his strength of movements. Urinary urge incontinence was also present.

In addition, his wife reported limb movement during sleep, although from his clinical history this was not compatible with REM Sleep Behavior Disorder [13]. His medical history also reported hypertension, anxiety-depression disorder, type 2 diabetes and chronic constipation. However, there was no mention of any family history of neurological disease.

On neurological examination the patient showed ataxic gait, with defined spasticity in the left lower limb, and asymmetric pendular movements of the upper limbs more evident on the right side*.* He maintained the Romberg position but with multidirectional oscillations, and was unable to perform tandem gait. Trunk and limb ataxia, especially on the left side, and adiadochokinesia of the left hand were evident. He was able to perform the finger-to-nose test and the heel-to-knee test with some difficulty on the left side. Brisk symmetrical deep tendon reflexes were detected with increase in the reflexogenic zone in the lower limbs and the upper left limb, but with absence of the Babinski sign. Mild hypokinesia was found in all 4 limbs, especially in the left foot.

Blood samples ruled out coeliac disease and vitamin E deficiency. Electromyography did not reveal lower motor neuron involvement; sensitive and motor nerve conduction studies were normal.

**Fig. 1:**
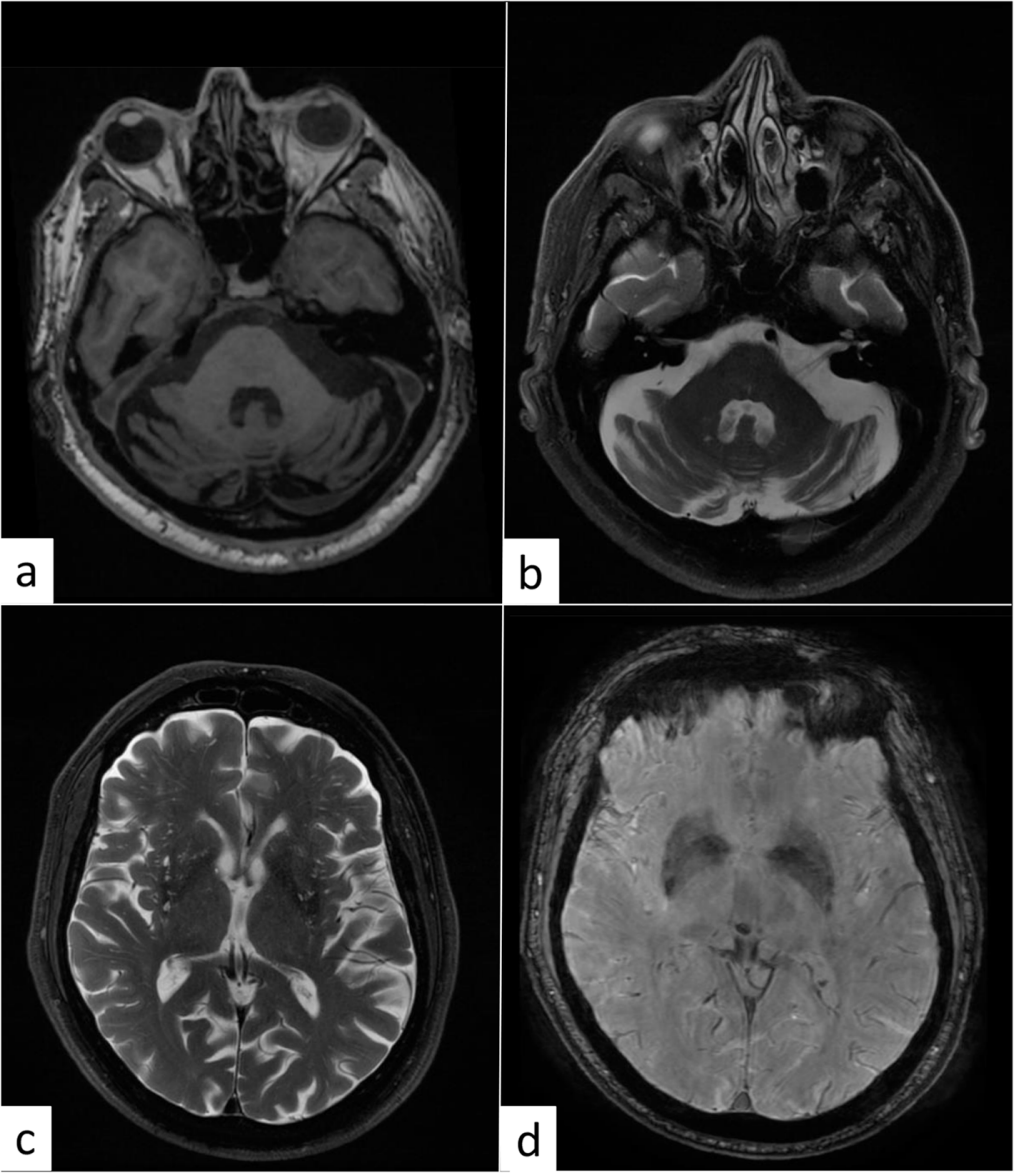
**a** T1 axial MRI scan shows bilateral hemispheric cerebellar atrophy **b** T2 axial MRI scan shows bilateral hemispheric cerebellar atrophy. **c** T2 MRI scan shows mild diffuse atrophy and enlarged perivascular space. **d** SWI MRI scan at the striatum level

MRI disclosed atrophy of the cerebellar hemispheres and vermis (Fig. 1a, b), and also revealed gliotic areas of the frontoparietal white matter bilaterally and dilated perivascular spaces at the basal ganglia level (Fig. 1c, d).

**Fig. 2:**
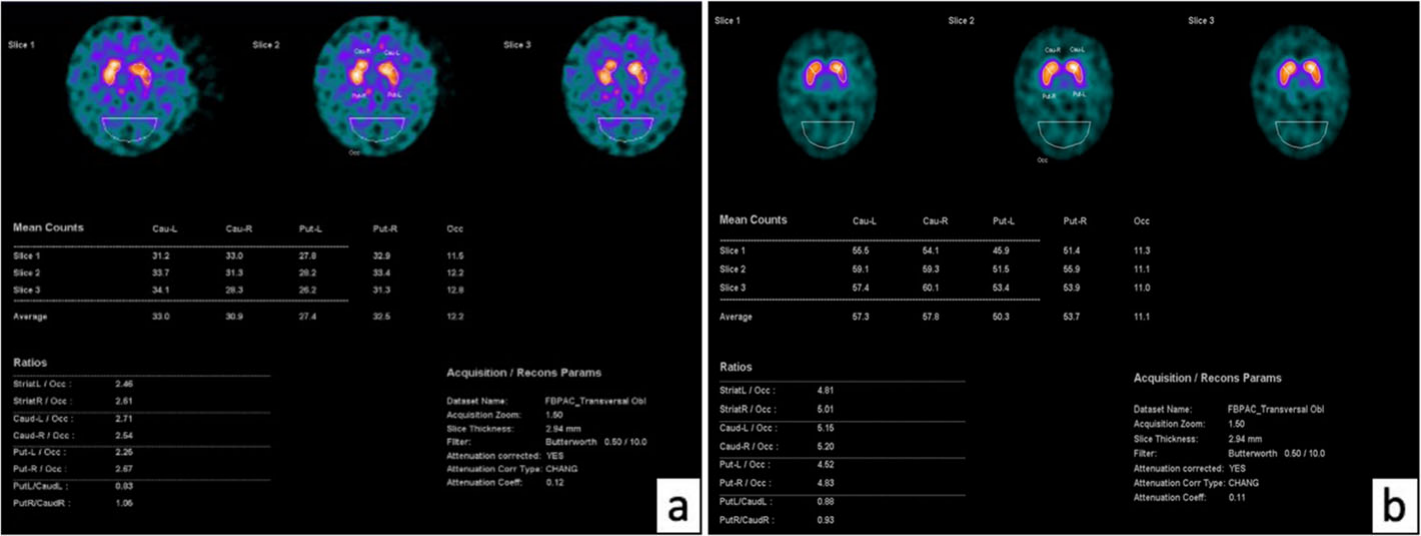
**a** Patient DAT-SCAN imaging shows a bilateral nigro-striatal degeneration. **b** DAT-SCAN imaging in a health control

DAT-SCAN imaging showed bilateral nigro-striatal degeneration (Fig. 2a, b), which oriented us towards a form of parkinsonism. Furthermore, the presence of ataxia concomitant with mild parkinsonism and pyramidal signs, in a patient with anamnestic presence of urinary dysfunction, led us to make a diagnosis of a possible MSA-C [14].

MSA mimicries should always be considered in the case of uncommon presentation of the disease [15–17]. Therefore, because of the atypical disease course (after 3 years from the onset of symptoms he was still walking without any support despite marked spasticity in the left leg), we conducted a next generation sequencing (NGS) analysis by Miseq illumine sequencer of all coding exons and related exon/intron junctions with SureSelectXT custom (Agilent Technologies) for the following genes: *FXN, ATM, SETX, TTPA, SACS, SIL1, NPC1, NPC2, ANO10, PHYH, OPA1, C10ORF2*, *SPG7*, *POLG*. Then we performed confirmation of variants by direct sequencing on DNA (Analyzer 3500, Applied biosystem). We also analyzed genes through Polymerase Chain Reaction analysis for SCA1, SCA2, SCA3, SCA6, SCA 7, SCA8, SCA12, SCA17, DLRPA, ATXN1, ATXN3, CACNA1A, ATXN7, ATXN80S, PPP2R2B, TBP, ATN1. A homozygous missense variant [c.1529C > T, p.(Ala510Val)] was found in the exon 11 of the *SPG7* gene. Although this variant can be detected up to 2–3% of normal individuals in large genomic databases, in the last decade its pathogenic role has been described [18].

The patient’s parents had lived in a small village of around 200 inhabitants, so we cannot exclude consanguinity.

## Discussion and conclusions

In this paper we reported a case of a patient with positive DAT-SCAN imaging and *SPG7* mutation. Moreover, the patient fulfilled the clinical-radiological criteria for a possible diagnosis of MSA-C.

The *SPG7* gene is constituted by 17 exons and encodes the protein paraplegin, a mitochondrial metalloprotease, located in the mitochondrial inner membrane [19]. It has a role in various mitochondrial processes such as mitochondrial protein quality surveillance and protein dislocation [20]. Mutations in mitochondrial DNA (mtDNA) are linked with parkinsonism because mithocondria can accumulate specifically in the dopaminergic neurons of the Substantia Nigra [21]. Low levels of mtDNA have been detected in Parkinson’s disease (PD) patients [22]. Animal model studies show an intense *SPG7* expression in the Purkinje cells, although only a moderate expression has been found in the striatum cells [23]. For these reasons, we suggest a possible involvement of the nigrostriatal pathways in patients with *SPG7* mutations.

A Spanish study investigated a cohort of 35 patients with *SPG7* mutations, 7 of whom exhibited Parkinsonism. Among them, the presence of parkinsonism was associated with gait ataxia (*n* = 6), spasticity (*n* = 4), extraocular movement dysfunctions (*n* = 3) or Pisa syndrome (*n* = 1) [24]. In a large cohort of 60 patients with *SPG7* mutations, the main presenting symptom was gait difficulty. Ataxia was present in 57% of all cases, thus it is considered the core symptom. A minor part of the patients presented dysarthria or nystagmus [5]. In MSA-C, ataxia of trunk and limbs, and other cerebellar symptoms, such as intention tremor and oculo-motor abnormalities (gaze-evoked or spontaneous nystagmus) are common [15]. However, patients with MSA-C frequently show parkinsonian features [25, 26].

Progressive and generalized autonomic failure often has an early onset in MSA. Erectile dysfunction is generally the first symptom, followed by urinary tract symptoms and cardiovascular autonomic failure. Autonomic symptoms can be considered a hallmark for differentiating MSA (especially sub-type C) from other causes of late or sporadic onset of ataxias [7], such as patients with *SPG7* mutations [12].

Considering brain imaging in patients with chronic ataxia, MRI shows three fundamental patterns of atrophy, namely, spinal atrophy, cortical cerebellar atrophy and olivopontocerebellar atrophy, with a substantial correlation between the MRI pattern and the different causes of inherited or acquired chronic ataxias [27].

In the Spanish study, the patients with *SPG7* mutations often presented cerebellar and brain atrophy on MRI (70 and 50% of the cases, respectively) [24]. Typical presentation of MSA-C on MRI includes atrophy of the putamen, pons, middle cerebellar peduncles and cerebellum [7]. Two specific findings.

frequently considered as hallmarks for MSA on T2-weighted images are the “putaminal hyperintense rim” a slit-like, marginal hyperintensity in the putamen [28, 29], and the “hot cross bun sign”, a cruciform pattern of hyperintensity in the basis pontis [30]. However, the hot cross bun sign may be present also in Spinocerebellar Ataxia (SCA) type 2, although patients tend to be younger and present a family history of ataxia [26].. Furthermore, generally, there is symmetric abnormal hypointensity of the putamen and caudate in a T2-weighted image, but it can also be present in progressive supranuclear palsy, Wilson’s disease, brain iron accumulation and in PD [31].

De la Casa-Fages and colleagues reported a case with *SPG7* mutation, positive at 123-Ioflupane single-photon emission computed tomography (SPECT) for bilateral nigrostriatal presynaptic denervation [24]. Together with our patient, this is one of the few cases described with positive DAT-SCAN and *SPG7* mutations.

Despite the lack of evidence of basal ganglia abnormalities on MRI in previous studies and in our MRI imaging [32], we observed nigrostriatal degeneration on the DAT-SCAN imaging, which could be related either to nigral degeneration or to dysfunction of the nigrostriatal synapses.

Patients with MSA-C generally show a decreased uptake of dopamine transporters in DAT-SCAN imaging and this could be considered a helpful tool for differentiating MSA-C from other causes of ataxia [27, 31]. However, in the literature, negative DAT-SCAN imaging has also been described in some patients with MSA [33], while a genetic cause of ataxia such as SCA2 and SCA3 may show a significant reduction in dopamine transporter levels in the striatum [34]. For these reasons, the DAT-SCAN result must be evaluated together with the clinical findings.

Ours is the first case report in the literature of a patient with *SPG7* mutation with nigrostriatal degeneration and a concomitant possible diagnosis of MSA-C. Our work underscores how increasingly important it is to perform genetic investigations in patients who present clinical features compatible with MSA-C, but which are atypical in terms of their disease course [15, 16]. Avoiding misdiagnosis in these patients is important not only for prognostic reasons, but also to find patients suitable for clinical trials and, hopefully, for gene therapy in the future.

## Data Availability

The data used during the current study are available from the corresponding author on reasonable request.

## Abbreviations

MSA: Multiple System Atrophy
MSA-P: Multiple system atrophy type P
MSA-C: Multiple system atrophy type C
HSP: Hereditary Spastic Paraplegias
SPG7: Spastic paraplegia type 7
CANVAS: Cerebellar ataxia, neuropathy and vestibular areflexia syndrome
MRI: Magnetic resonance imaging
DAT-SCAN imaging: Dopamine Transporter Scan imaging
mtDNA: Mitochondrial DNA
PD: Parkinson’s disease
SCA: Spinocerebellar Ataxia
SPECT: Single-photon emission computed tomography
